# A new remarkable Early Cretaceous nelumbonaceous fossil bridges the gap between herbaceous aquatic and woody protealeans

**DOI:** 10.1038/s41598-023-33356-z

**Published:** 2023-06-02

**Authors:** William Vieira Gobo, Lutz Kunzmann, Roberto Iannuzzi, Thamiris Barbosa dos Santos, Domingas Maria da Conceição, Daniel Rodrigues do Nascimento, Wellington Ferreira da Silva Filho, Julien B. Bachelier, Clément Coiffard

**Affiliations:** 1 Departamento de Paleontologia e Estratigrafia, Universidade Federal do Rio Grande do Sul (UFRGS), Rio Grande do Sul, Ave. Bento Gonçalves 9500, Porto Alegre, 91501-970 Brazil; 2 Abteilung Museum für Mineralogie und Geologie, Senckenberg Naturhistorische Sammlungen Dresden, Königsbrücker Landstrasse. 159, D–01109 Dresden, Germany; 3 Universidade Regional do Cariri (URCA), St. Cel. Antônio Luíz 1161, Museu de Paleontologia Plácido Cidade Nuvens, Crato, Ceará 63105-010 Brazil; 4 Departamento de Geologia, Universidade Federal do Ceará (UFC), Campus do Pici – 912, Fortaleza, Ceará 60440-554 Brazil; 5 Structural and Functional Plant Diversity Group, Dahlem Centre of Plant Sciences, Institute of Biology, Freie Universität Berlin, Altensteinstrasse 6, 14195 Berlin, Germany

**Keywords:** Palaeontology, Taxonomy

## Abstract

Dating back to the late Early Cretaceous, the macrofossil record of the iconic lotus family (Nelumbonaceae) is one of the oldest of flowering plants and suggests that their unmistakable leaves and nutlets embedded in large pitted receptacular fruits evolved relatively little in the 100 million years since their first known appearance. Here we describe a new fossil from the late Barremian/Aptian Crato Formation flora (NE Brazil) with both vegetative and reproductive structures, *Notocyamus hydrophobus* gen. nov. et sp. nov., which is now the oldest and most complete fossil record of Nelumbonaceae. In addition, it displays a unique mosaic of ancestral and derived macro- and micromorphological traits that has never been documented before in this family. This new Brazilian fossil-species also provides a rare illustration of the potential morphological and anatomical transitions experienced by Nelumbonaceae prior to a long period of relative stasis. Its potential plesiomorphic and apomorphic features shared with Proteaceae and Platanaceae not only fill a major morphological gap within Proteales but also provide new support for their unexpected relationships first suggested by molecular phylogenies.

## Introduction

The origin and early radiation of flowering plants are still unclear and remain one of the most enigmatic themes concerning the evolutionary history of vascular plants as a whole^1^. Although the fossil record may help to reduce morphological gaps between living taxa, it is inherently incomplete and may thus miss important evolutionary transitions. Such gaps in evidence for documenting plant evolution can be interpreted either as a consequence of a period with higher evolutionary rates over a short timescale or lower rates of evolution and relative stasis over a long timescale^2^. However, the scarcity of earliest and succeeding forms in fossil records can be also caused by preservational biases, undersampling and low intensity of investigation. Numerous studies of middle paleolatitude floras have significantly reduced the knowledge gaps on Early Cretaceous angiosperms, while those from paleotropical areas, where allegedly flowering plants started to diversify, are still in need of more investigations, especially regarding macrofossils of eudicots, the most diverse angiosperm clade^3,4^. The knowledge gaps on macrofossils in the Early Cretaceous tropics contrast with the substantial and comparatively well-studied microfossil record^5,6^.

The first reports of eudicots date back to the middle Early Cretaceous from circumtethyan areas, i.e., northern Gondwanan and southern Laurasian regions, and suggest that their origin and center of diversification were in northern Gondwana^5,7^. These reports are mainly based on their distinctive tricolpate pollen^7^, but also on macro- and mesofossils thought to be related to basal lineages^8,9^. Among the pieces of evidence, some records are related to Ranunculales and Proteales^8,9^, which are the two earliest diverging lineages of eudicots according to molecular analyses^10^. However, in contrast to the extant families of Ranunculales, there are huge differences and remarkable gaps in gross morphologies between those of Proteales, i.e., Sabiaceae, Nelumbonaceae, Platanaceae, and Proteaceae^9^. Their close phylogenetic relationship was thus not suspected before advances in molecular phylogenetics^9–11^, and potential morphological synapomorphies supporting their shared common ancestry started to be ascertained thereafter^12,13^. In addition, all these families occur in Cretaceous floras and Nelumbonaceae, Platanaceae, and Proteaceae particularly stand out by their relative abundance and richness in the fossil record^9^. Unfortunately, few intermediate forms linking the morphologies of these families have been found, and it seemed unlikely that they would be found since Nelumbonaceae and Platanaceae have undergone a long period of relative morphological stasis^14,15^.

Representatives of Nelumbonaceae are thought to have remained more or less similar in terms of their gross morphologies from the late Early Cretaceous to the present day, and they are relatively diverse not only through geologic time but also across the world. For instance, *Nelumbites* Berry is found in the middle Albian of Kazakhstan^16^, the upper Albian of Siberia^17^, and the middle and upper Albian Potomac Group of Virginia (USA)^18,19^, and the latter was confirmed its nelumbonaceous relationship by a cladistic analysis^19^. There are also *Exnelumbites* Estrada-Ruiz, Upchurch, Wolfe et Cevallos-Ferriz from the Campanian to the Maastrichtian of Mexico^20^, and *Nelumbago* McIver et Basinger and *Paleonelumbo* Knowlton documented from the Paleocene of Canada^21^ and Colorado (USA)^22^, respectively. Although numerous reports coming from Africa, Asia, Europe, and North and South America are attributed to *Nelumbo* Adans., most of them do not bear synapomorphies of the crown group and need a taxonomic reevaluation^15,23–25^. These fossils suggest that, mainly from the Late Cretaceous onwards, Nelumbonaceae, including *Nelumbo*, have had a nearly worldwide distribution, mostly in the Northern Hemisphere. Today, the genus has a relictual disjunct distribution with two extant species, i.e., wild populations of *N. lutea* (Willd.) Pers. in southern North America and *N. nucifera* Gaertn. in southern Asia to Australasia^25,26^.

In this paper, we describe a new fossil-genus and fossil-species of Nelumbonaceae from the Crato Formation of Brazil. This new macrofossil-taxon from the middle Early Cretaceous not only represents the earliest occurrence of this family known to date but also displays a mosaic of traits finally bridging the morphological gap between Nelumbonaceae and their woody sister clade consisting of Platanaceae and Proteaceae.

## Details of specimens

### Geological setting and age

The sedimentary Araripe Basin (NE Brazil) includes several lithological units among which the Santana Group stands out in yielding fossil lagerstätten, especially that from the Crato Formation^27,28^. The Crato Formation mainly represents a lacustrine paleoenvironment documented by laminated limestones^29^. In these lacustrine rocks, especially the stratigraphically youngest C6 carbonate unit, countless fossils of vertebrates, invertebrates, and plants are found^28,29^. Such abundance of fossils and their often remarkable preservation have led to the designation of the Crato Formation as a Konservat-Lagerstätte^28^. Its scientific value is demonstrated by the fact that it contains one of the currently best records for understanding mid-Early Cretaceous low-latitude paleo-ecosystems^28,30^. The age of the Crato Formation has usually been considered to be upper Aptian^31,32^, but there is no longer a consensus since new age estimates for the Santana Group, together with an updated concept and position for the international chronostratigraphic unit Aptian, suggest that at least a lowermost Aptian age (ca. 121 Ma) is more plausible (see in Methods)^29,33–36^. Furthermore, the usual lack of information on the detailed lithostratigraphic positions of fossils collected from the C6 unit of the Crato Fossil Lagerstätte raises the question of whether particular organisms existed simultaneously or not since the laminated limestone section represents a substantial time interval (200 to 500 ka–see estimates of Neumann et al.^29^). The latter fact complicates reconstructions of the paleoecological context of certain fossil biotas from the lagerstätte.

### The Crato Formation flora

The macrofossil assemblage of the Crato Formation includes a few spore-bearing plants but it is dominated by seed plants (i.e., gymnosperms and angiosperms)^30^. Angiosperms show a notably high taxonomic diversity and wide paleoecological spectrum even though not all fossil elements have been formally described yet^37^. Mesangiosperms make up the bulk of described fossils, including magnoliids^38–41^, monocots^6,42^, and eudicots^8^. By contrast, only a few records were assigned to the ANA grade, specifically Nymphaeales^43,44^. In addition, the angiosperm pollen records are mainly represented by monocolpate and tricolpate forms and may support the presence of chloranthoid, magnoliid, monocot, and eudicot taxa^28,32,37^.

## Results

- Angiospermae Lindley (P.D.Cantino et M.J.Donoghue).
- Eudicotyledoneae M.J.Donoghue, J.A.Doyle et P.D.Cantino.
- Order Proteales Juss. ex Berchtold et J.Presl.
- Family Nelumbonaceae A.Rich.
- *Notocyamus* Gobo, Coiffard, Bachelier, L.Kunzmann, Conceição et Iannuzzi, gen.nov.

### Generic diagnosis

Aquatic angiosperm with herbaceous rhizomatous growth habit, with adventitious roots and long-petiolate, alternate simple leaves. Leaf with marginal petiole attachment, unlobed and untoothed margin, and distinctly papillate epidermis with anomocytic and (brachy-) paracytic stomata on both sides. Primary venation palinactinodromous, distally bifurcating, with agrophic veins. Major secondary venation festooned brochidodromous. Fruit solitary, terminal, and aggregate, derived from an apocarpous gynoecium with fruitlets embedded in an enlarged ellipsoidal receptacle, attached to a long woody peduncle. Fruitlets inserted in pits and free from the receptacle.

### Etymology

*Noto* is derived from *notos* from Ancient Greek νότος (south) and *cyamus* from Ancient Greek κύαμος (bean), in reference to the Egyptian “bean” described by Theophrastos of Eresos, which was probably a lotus seed.

### Plant fossil names registry number

PFN003134

### Type species

*Notocyamus hydrophobus* Gobo, Coiffard, Bachelier, L. Kunzmann, Conceição et Iannuzzi, sp. nov.

### Remarks

The main diagnostic characters of *Notocyamus* gen. nov. are the palinactinodromous leaf venation, in which lateral primary veins branch successively rather than from one point, and the secondary xylem in the peduncle, which are distinct from any living *Nelumbo* or any fossil-genus in the family.

***Notocyamus hydrophobus*** **Gobo, Coiffard, Bachelier, L. Kunzmann, Conceição et Iannuzzi, sp. nov.**

### Species diagnosis

Lamina noto- to mesophyllous in size with L/W ratio ca. 1, chartaceous, obovate to elliptical, with obtuse and rounded to slightly cordate base and obtuse and rounded to straight-sided apex. Interior secondaries and intersecondary veins present. Tertiary venation mixed percurrent, predominantly opposite. Quaternary venation mixed percurrent, mostly alternate. Quinternary veins regular reticulate. Areolation five- to six-sided. Freely ending veinlets absent. Marginal ultimate venation looped.

### Etymology

*hydrophobus* is derived from Ancient Greek ὑδροφόβος meaning fearing water, in relation to the epidermis structure similar to the superhydrophobic epidermis of living lotus.

### Holotype

**Figure 1:**
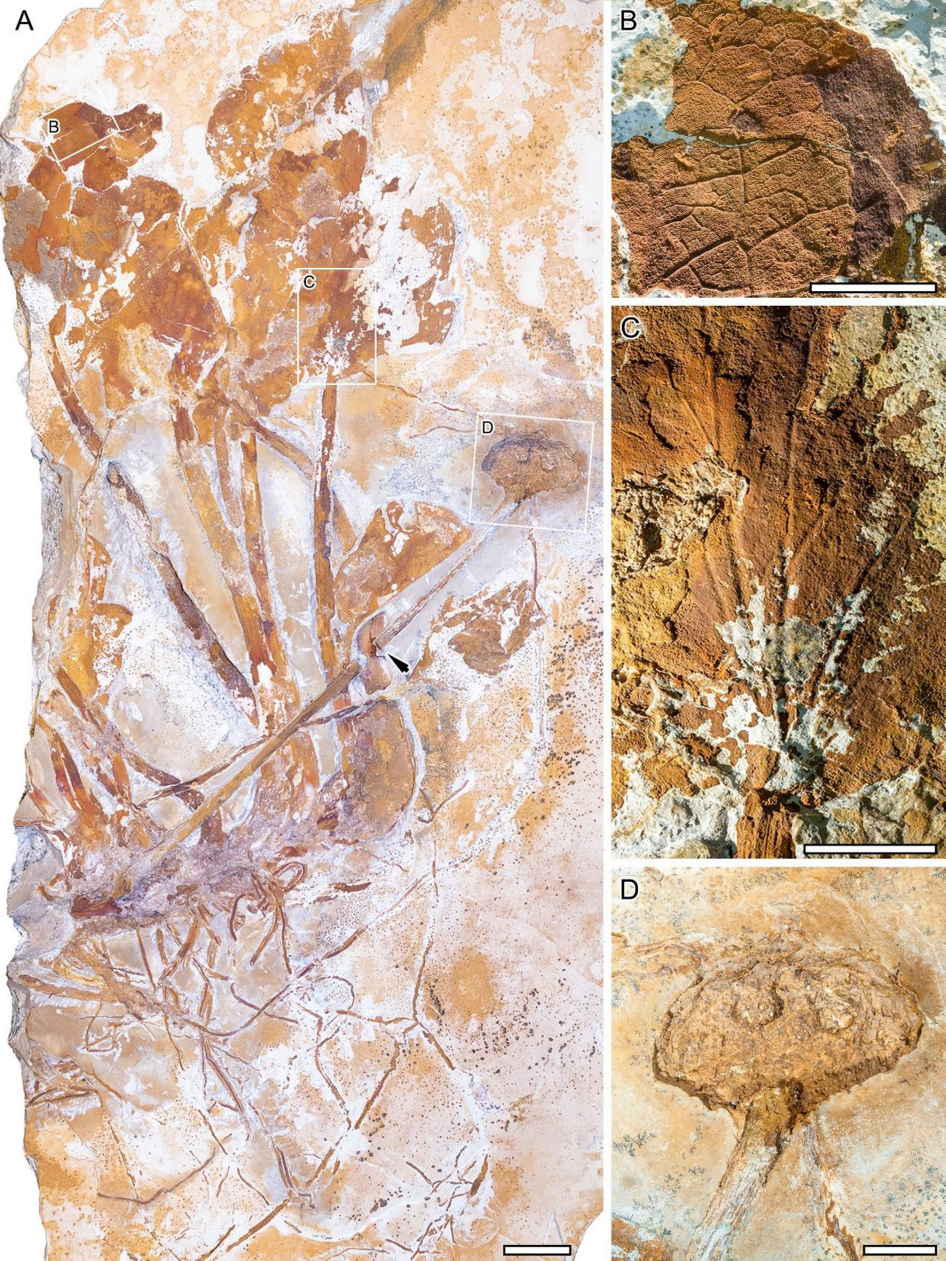
*Notocyamus hydrophobus* gen. nov. et sp. nov. (holotype, MB. Pb. 2002/1047). (**A**) Overview of the whole plant, with roots, rhizome, leaves, and aggregate fruit in organic connection. A black arrow points to the part of the peduncle used to make the thin sections (see Figs. 5 and 6). (**B**) Details of higher-order venation. (**C**) Close-up of palinactinodromous venation and the marginal lamina attachment. (**D**) Close-up on the enlarged receptacle showing two globose fruitlets (presumed nutlets). Scale bars: 1 cm.

MB.Pb. 2002/1047 (repository: Museum für Naturkunde—Leibniz Institute for Evolution and Biodiversity Science, Berlin, Germany), Fig. 1.

### Paratype

**Figure 2:**
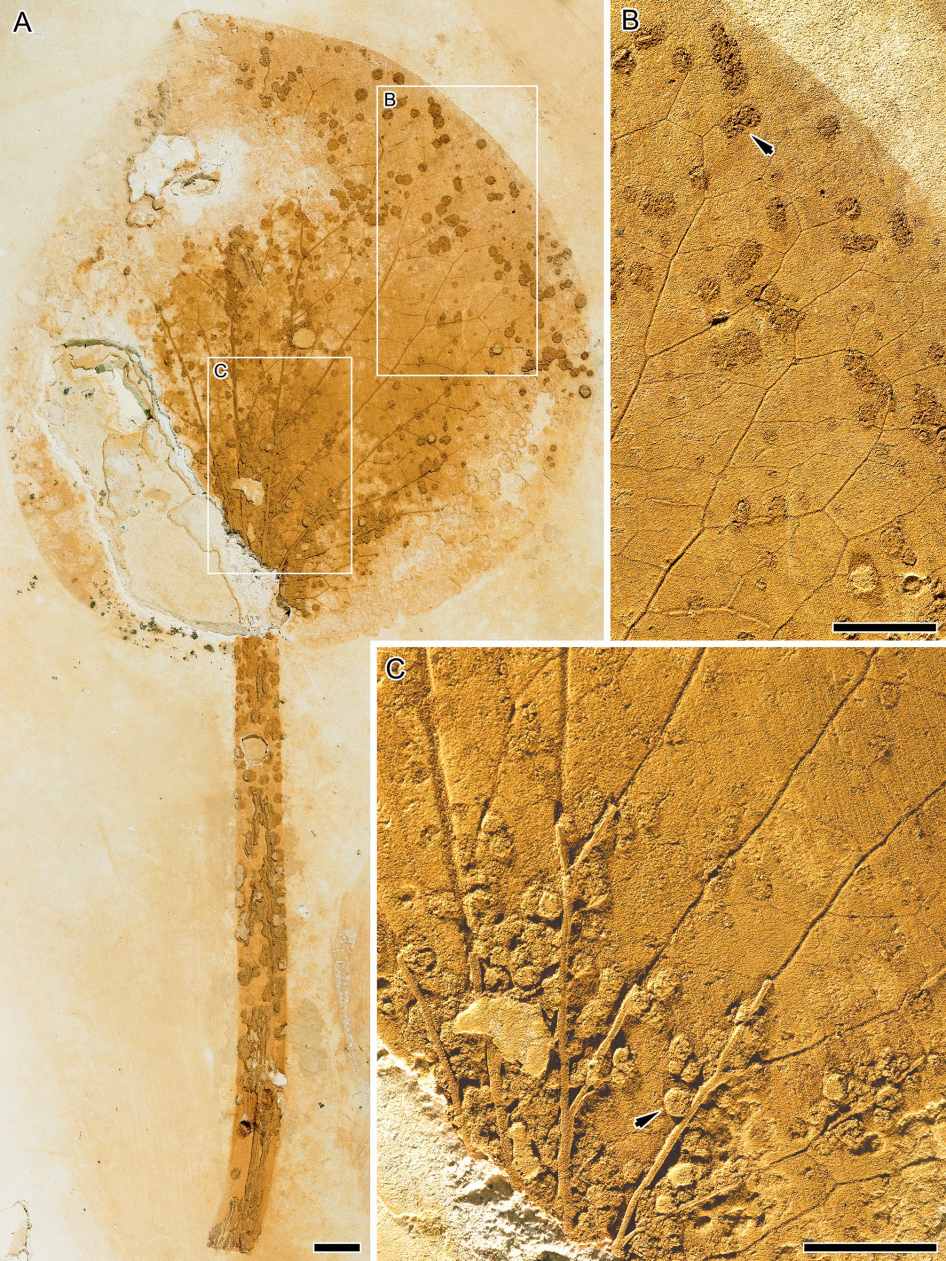
*Notocyamus hydrophobus* gen. nov. et sp. nov. (paratype, SMF SM.B 16.522). (**A**) Isolated leaf. (**B**, **C**) Close-ups of the pattern of venation. Note that abundant structures interpreted as galls occur along the lamina and petiole (arrows point to some of them). Scale bars: 5 mm.

SMF (SM.B 16.522) (repository: Senckenberg Forschungsinstitut und Naturmuseum, Frankfurt am Main, Germany), Fig. 2.

### Plant fossil names registry number

PFN003135

### Type horizon and locality

Opencast pit(s) southwest of Nova Olinda, Ceará, Brazil. Lower Cretaceous (uppermost Barremian?/lowermost Aptian), C6 limestone horizon, Crato Formation, Santana Group, Araripe Basin.

### Remarks

Vegetative structures are preserved as reddish-brown replacement fossils and/or impressions with or without a thin iron oxide covering layer. Tissues of the peduncle are also replaced by iron oxide, while the receptacle is a mold with two casts of fruitlets. Clastic material is especially abundant on the rhizome of the holotype, and also in the petiole of the paratype.

### Description and remarks

The holotype is about 50 cm long and 25 cm wide and consists of a rhizome bearing about 30 roots, 13 leaves, and a single aggregate fruit with two visible fruitlets embedded in an enlarged receptacle (Fig. 1). The rhizome is unbranched and about 15 cm long and 1–2 cm wide. The roots are adventitious and up to at least 20 cm long and 0.3 cm wide.

**Figure 3:**
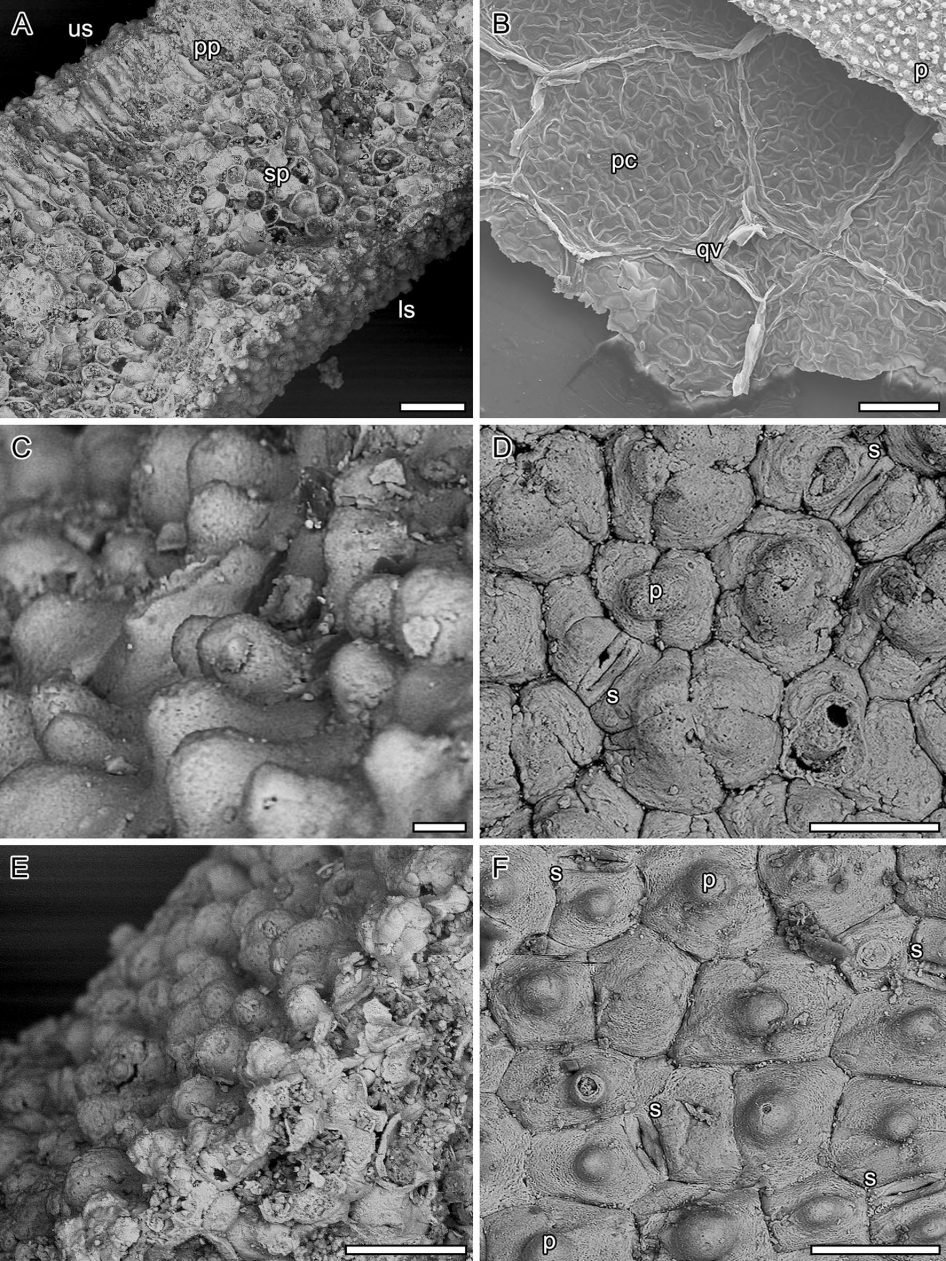
*Notocyamus hydrophobus* gen. nov. et sp. nov. (holotype, MB. Pb. 2002/1047). (**A**–**F**) SEM images of leaf. (**A**) Leaf cross-section showing differentiation of the mesophyll into upper palisade parenchyma with elongate and cylindrical cells, and lower spongy parenchyma with irregular and globose cells. (**B**) Quinternary leaf veins forming 5–6 sided polygons (areoles) and delimiting areas with undulate walls interpreted as being from the parenchyma (in transversal section). (**C**–**D**) Upper epidermis. (**C**) Papillae on the adaxial leaf surface. (**D**, **F**) Stomata and their neighboring epidermal cells bearing papillae (**E**–**F**) Lower epidermis. (**E**) Papillae on the abaxial leaf surface. Abbreviations: us—upper surface; pp—palisade parenchyma; sp—spongy parenchyma; ls—lower surface; pc—parenchyma cells; qv—quinternary veins; p—papilla; s—stoma. Scale bars: **A**, **B** = 100 µm; **C** = 10 µm; **D**, **E**, **F** = 50 µm.

The leaves are petiolate, alternate and simple. Petioles are 12–20 cm long by 0.5–1 cm width. The position of the lamina attachment to the petiole is marginal. Laminar sizes are noto- to mesophyllous, 60 to 95 mm long, and about 60 to 100 mm wide. Laminar shapes are obovate to elliptical, slightly medially asymmetrical to symmetrical, with a length/width (L/W) ratio of about 1. The lamina texture is likely chartaceous. Leaves are unlobed with untoothed margins, obtuse and rounded to slightly cordate bases, and obtuse and rounded to straight-sided apices. The primary venation is palinactinodromous with five primary veins running decurrently basally and bifurcating once or twice distally (Figs. 1C and 2). Naked basal veins are absent, and agrophic veins are present. The midvein is around 0.5–1 mm wide while the lateral primaries are about 0.3–0.8 mm wide. The major secondary venation consists of festooned brochidodromous veins (Figs. 1B and 2B), which are about 0.08–0.14 mm wide. Interior secondaries and intersecondaries are present. The course of the minor secondaries is simple brochidodromous, and a perimarginal vein is absent. The spacing of secondary veins decreases gradually toward the lamina base. Their attachment to the primaries is excurrent and forms a uniform acute angle (about 40°) to the primary veins. Intercostal tertiary veins are mostly opposite percurrent, with convex to sinuous courses (Figs. 1B and 2B). They are obtuse to nearly perpendicular to the midvein with angles decreasing exmedially and are about 0.06–0.09 mm wide. Exterior tertiaries form loops. The quaternary veins are mostly alternate percurrent and about 0.04–0.07 mm wide (Figs. 1B and 2B). Quinternary veins are regular reticulate and about 0.02–0.03 mm wide (Figs. 1B and 3B). The areolation is well developed, 5- to 6-sided (Fig. 3B). Freely ending veinlets are absent. Marginal ultimate venation is looped (Figs. 1B and 2B). Vein density ranges between 7.8–9.9 mm^−1^ (mean: 9 mm^−1^).

**Figure 4:**
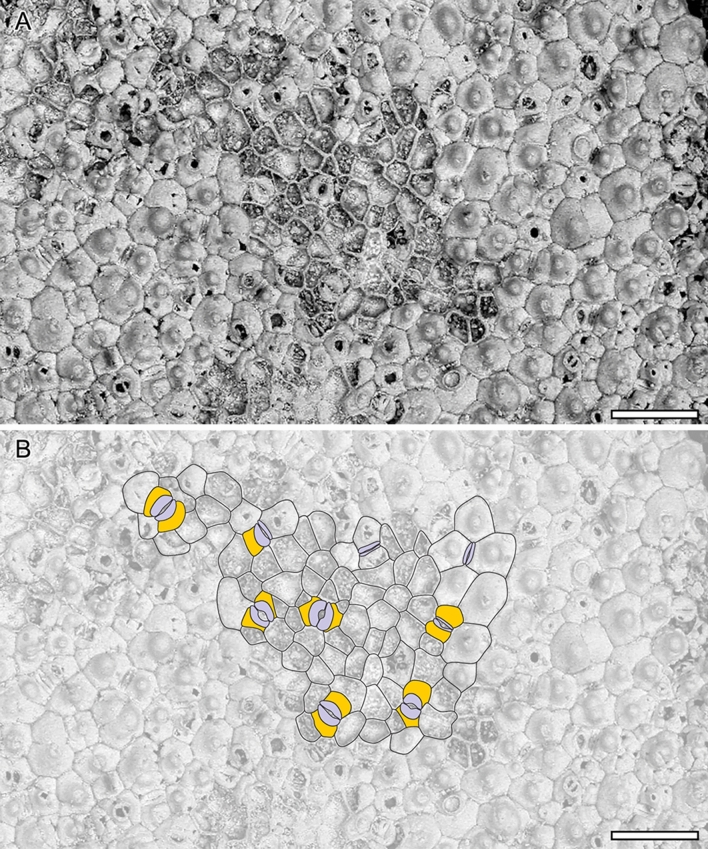
*Notocyamus hydrophobus* gen. nov. et sp. nov. (holotype, MB. Pb. 2002/1047). (**A**) SEM image of upper leaf epidermis showing abraded zones without periclinal walls. (**B**) drawing of epidermal cells and their paracytic and anomocytic stomata. The guard cells are colored in blue and subsidiary cells in orange. Scale bars: 100 µm.

The leaves are bifacial and 0.5 mm thick (Fig. 3A). The upper epidermis is uniseriate and papillate, and 20–25 µm thick. Epidermal cells are penta- to heptagonal, 30–70 µm long and 25–45 µm wide, with 3–4 µm thick straight anticlinal cell walls and a single papilla per cell (Fig. 3A,D). These papillae are 10–25 µm in diameter and 15–20 µm high (Fig. 3C,D). The lower epidermis is also uniseriate, papillate, and 20–30 µm thick. Its cells are essentially similar in shape to those of the upper epidermis, but slightly smaller and 30–50 µm long and 25–30 µm wide (Fig. 3A,E,F). They also display 3–5 µm thick straight anticlinal cell walls and a single well-developed papilla per cell. However, these papillae are also slightly smaller than those of the upper epidermis and 8–25 µm in diameter and 10–15 µm high (Fig. 3E,F). The leaves are amphistomatic with a stomatal density (SD) of about 60–150 mm^−2^ adaxially, and 100–120 mm^−2^ abaxially. The stomata are usually 30–35 µm long and 8–30 µm wide, with 15–25 µm long apertures randomly oriented. When the outer periclinal walls of the epidermis are abraded, their guard cells are often kidney-shaped (Fig. 4A,B). The cells in contact with the guard cells are variously shaped, rectangular-polygonal and often asymmetrically in outline, with straight anticlinal cell walls. If these lateral cells belong to the stomatal complexes, the complexes would be (brachy-) paracytic but otherwise anomocytic. In the non-abraded case, the two lateral neighboring cells often partly overlap the guard cells, resulting in a narrow elliptical outline of the stomata in outer surface view (Fig. 3D,F). Compared to the abraded case, the rectangular lateral neighboring cells are often not clearly distinguishable from the ordinary epidermal cells that are characterized by a single papilla on the periclinal walls (Figs. 3D,F and 4). The mesophyll displays a clear dorsiventral organization with an upper/adaxial palisade parenchyma and a lower spongy parenchyma (Fig. 3A). The single cell-layered palisade parenchyma is about 100–150 µm thick, and comprises cylindrical cells with 15–25 widths which display 1–2 µm thick primary walls. The spongy parenchyma is about 300–350 µm thick and consists of more or less globose to irregularly shaped cells that are 20–60 µm in diameter, with 3–4 µm thick primary walls. However, most of these cells are collapsed and their organization is not clear even though some lacunae can be observed.

The peduncle of the fruit is 22.5 cm long and 4 mm wide and ends with the hollow mold of the fruit. The fruit is formed by an enlarged ellipsoidal receptacle, which is 2.5 cm long and 4 cm wide (Fig. 1D). It exhibits two globose fruitlets that are about 0.5 cm in diameter and embedded in distinct receptacular pits. Although the distribution of pits on the receptacle remains unclear, the fruit is interpreted as an aggregate derived from an apocarpous gynoecium, where each carpel develops into a fruitlet, likely a nutlet, with an estimated volume of 65 mm^3^ on average. No traces or remains of a perianth or other floral/fruit structures are observed below or around the receptacle.

**Figure 5:**
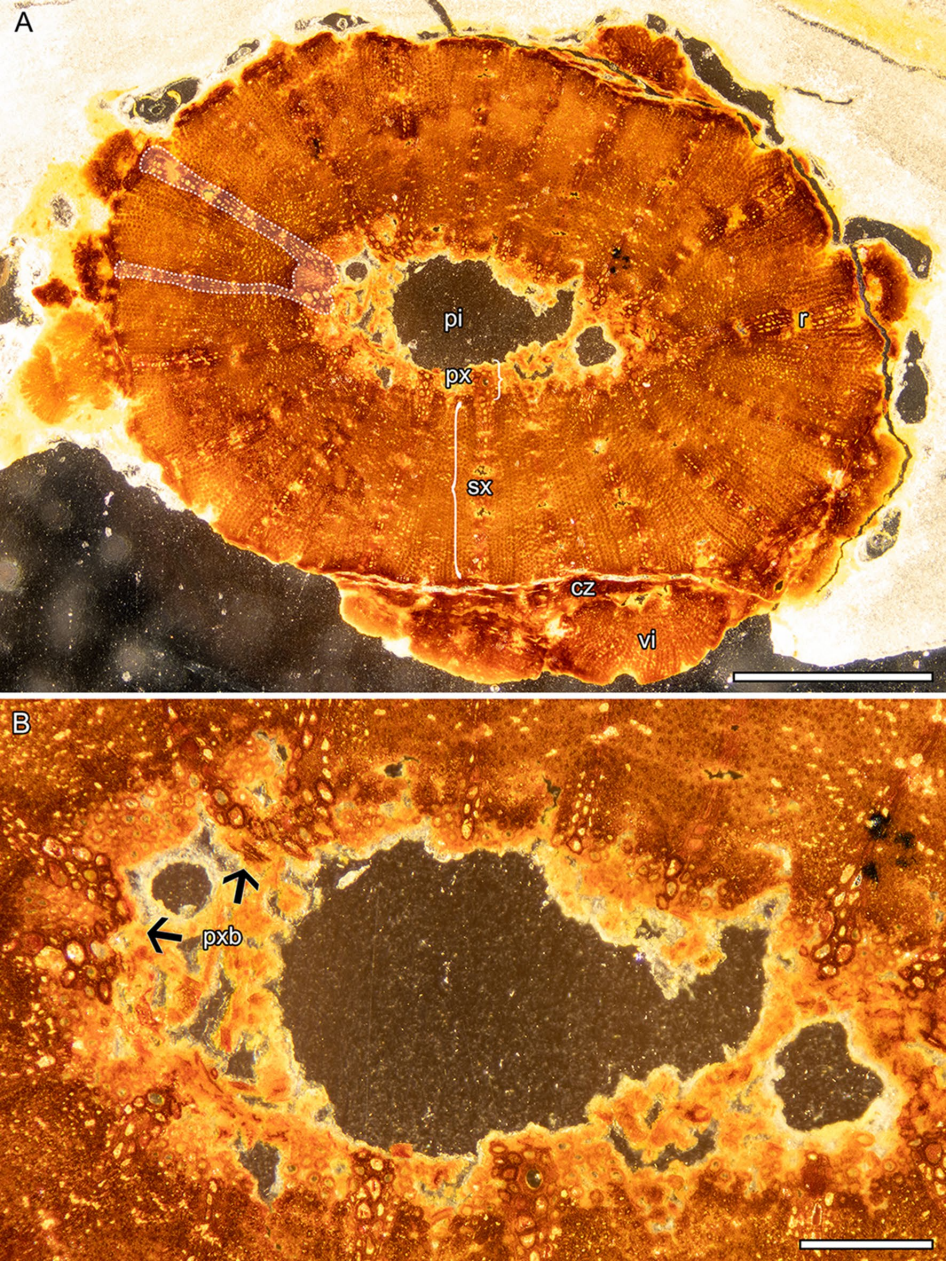
*Notocyamus hydrophobus* gen. nov. et sp. nov. (holotype, MB. Pb. 2002/1047). (**A**–**B**) Transversal section of the peduncle. (**A**) Overview displaying homoxylic wood with abundant fibers interspersed with parenchymatous rays. The dashed outline shows the rays connecting with primary xylem that form bundles near the pith. On the periphery, remains of the cambial zone occur in a discontinuous layer that is followed by new vascular increments. (**B**) Close-up of region near pith showing the primary xylem bundles and their connection with the rays. Abbreviations: pi—pith; px—primary xylem; sx—secondary xylem; r—ray; cz—cambial zone; vi—vascular increments; pxb—primary xylem bundles. Scale bars: **A** = 1 mm; **B** = 250 µm.

**Figure 6:**
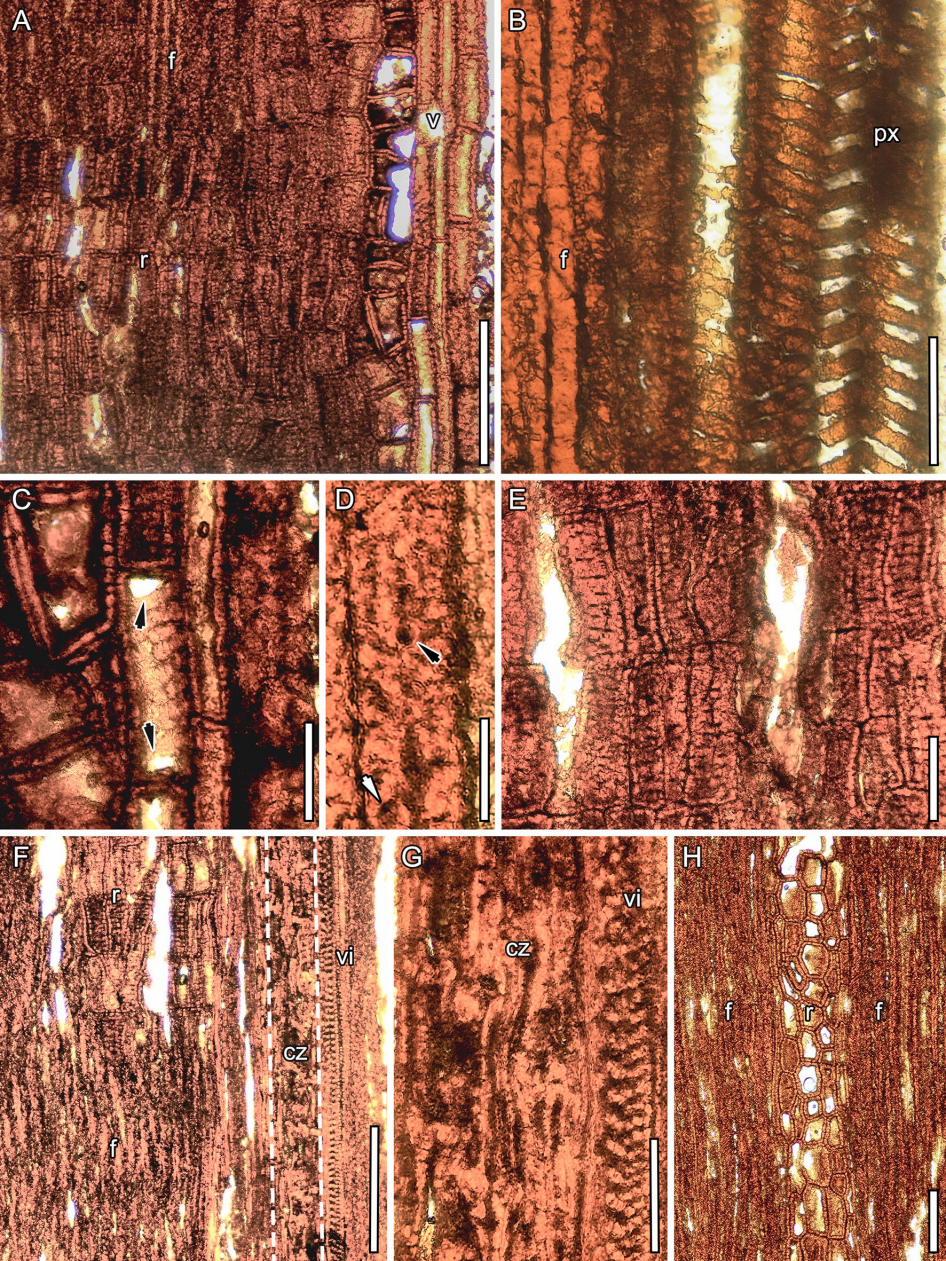
*Notocyamus hydrophobus* gen. nov. et sp. nov. (holotype, MB. Pb. 2002/1047). Radial (**A**–**G**) and tangential (**H**) sections of the peduncle. (**A**) Overview of region adjacent to the pith (to the far right) showing vessels in the xylem followed by the secondary xylem with ray and fiber cells (to the far left). (**B**) Primary xylem cells with helical thickenings and fibers of secondary xylem. (**C**) A vessel element with scalariform-like pits and simple perforation plates (arrows). (**D**) Close-up of a fiber showing simple (black arrow) and slit-like pits (white arrow). (**E**) Ray cells with slit-like pit apertures. (**F**) Overview of region near cambial zone (dashed outline) showing fiber and ray cells in the secondary xylem followed by the layer that gives rise to the new vascular increments. (**G**) Fusiform and compressed cells from the cambial zone followed by tracheary elements with scalariform-like pits. (**H**) Ray cells in between fibers of secondary xylem. Abbreviations: v—vessels; px—primary xylem; r—ray; f—fiber; cz—cambial zone; vi—vascular increments. Scale bars: **A**, **F** = 200 µm; **B**, **C**, **E**, **G** = 50 µm; **D** = 25 µm; **H** = 100 µm.

The peduncle shows a pith surrounded by primary xylem bundles and secondary homoxylic (vesselless) xylem without growth rings, and is surrounded by a (peripheral) discontinuous layer interpreted as the remains of the cambial zone (Figs. 5 and 6).

The pith is ovate and 0.6–1.5 mm in diameter (Fig. 5). No tissue is preserved at the center of the pith, but it is unclear whether it was originally hollow or not.

The inner ring of primary xylem comprises ca. 18(19?) xylem bundles, with cells that are unevenly isodiametric and have a 13 to 60 µm wide diameter and a 4–10 µm thick wall (Fig. 5B). Walls of the xylem cells usually have helical and scalariform thickenings (Fig. 6B). Vessel elements are present and appear closer to the pith. They are usually rectangular, upright to drum-shaped, about 40–286 µm long and 17–60 µm wide, and although they are sometimes poorly preserved, scalariform-like pits occur in their side walls and simple perforation plates typically occur in their end walls (Fig. 6A,C).

The homoxylic wood (secondary xylem) consists predominantly of cells interpreted as fibers separated by 19 parenchymatous, homocellular, and usually multiseriate (rarely uniseriate) rays that are about 30–120 µm wide (Fig. 5A). In contrast to the primary xylem cells, the fibers appear more uniform in diameter (15–30 µm), with a 5–10 µm thick wall, a very small lumen, and simple and slit-like pits (Figs. 5A, 6D). Ray cells are also usually more regular, rectangular, and upright, and have a 3–8 µm thick wall with slit-like pit apertures (Fig. 6A,E,F). Near the pith, they connect with the primary xylem (Fig. 5).

The cambial zone is very poorly preserved. However, it comprises compressed fusiform and elongated cells outside the secondary xylem, from which arise new vascular increments with tracheary elements characterized by scalariform-like pits, including fibers with simple pits (Figs. 5A and 6F,G). These vascular increments are probably derived from anomalous secondary growth.

**Figure 7:**
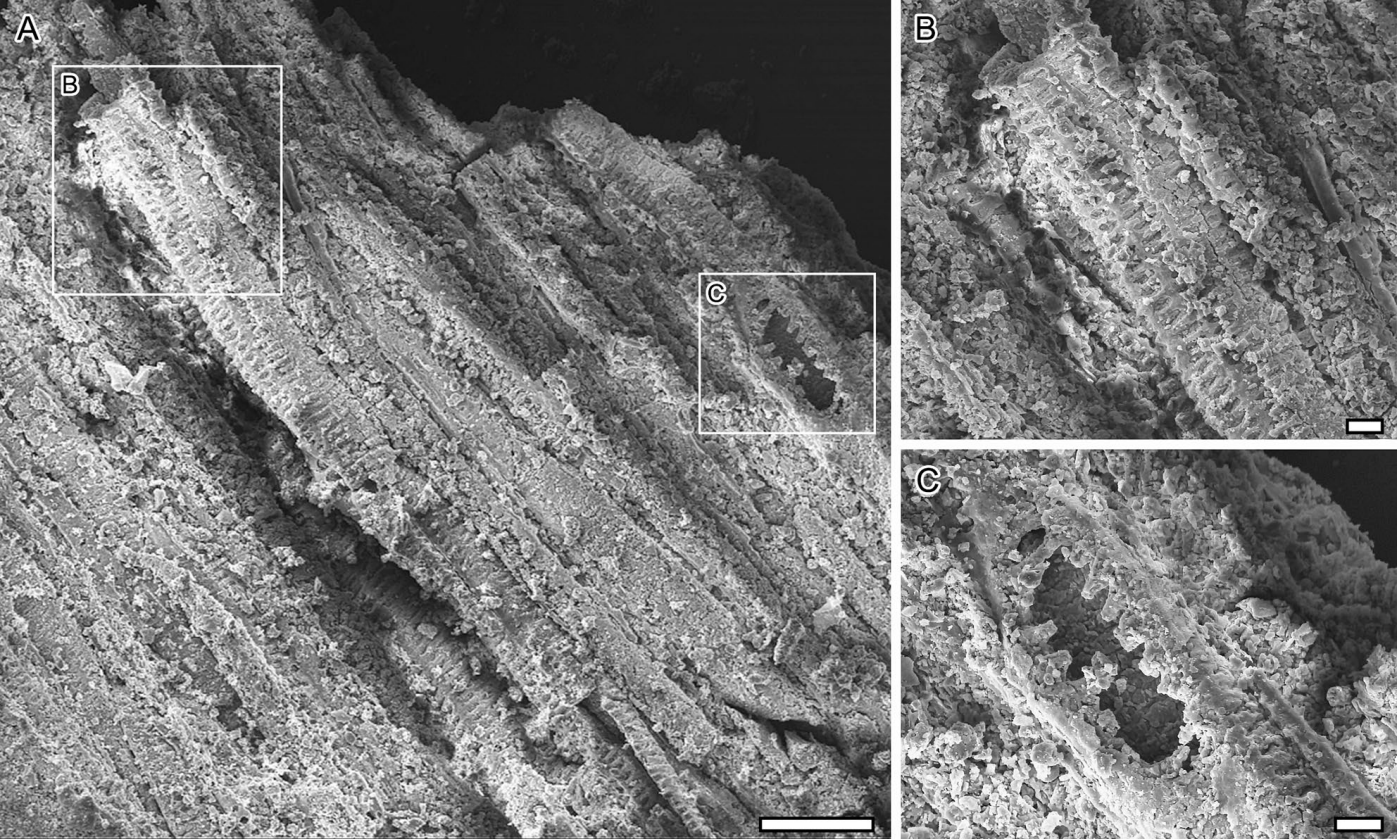
*Notocyamus hydrophobus* gen. nov. et sp. nov. (holotype, MB. Pb. 2002/1047). (**A**–**C**) SEM images of longitudinal views of the petiole. (**A**) Overview of vessel elements. (**B**) Close-up of a vessel element showing the scalariform pits. (**C**) Close-up of a scalariform perforation plate. Scale bars: **A** = 100 µm; **B**, **C** = 20 µm.

The anatomy of the petiole also includes vessel elements, which are 65–75 µm in diameter and have 5 µm thick secondary walls with scalariform pits and scalariform perforation plates (Fig. 7). However, in contrast to that the peduncle, it also has abundant aerenchymatous channels (seen as lacunae) and lacks secondary growth.

### Plant–insect interactions

A single insect herbivory trace identified as Damage Type (DT) 125 linked to the Functional Feeding Group (FFG) Galling was found in *Notocyamus* (Fig. 2), following the classification guidelines of Labandeira^45^. The galled tissue is thick, circular, and discoidal in shape, and the bodies can be aggregated to one another or isolated along the leaf and petiole. They are about 0.6–1.4 mm long and 0.7–1.44 mm wide.

### Phylogenetic analysis

**Figure 8:**
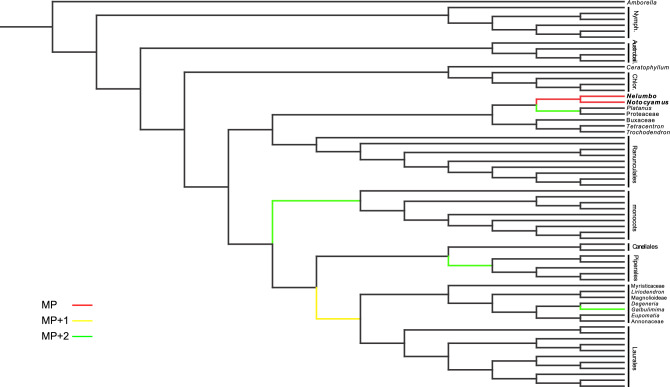
Cladistic analysis of the position of *Notocyamus hydrophobus* gen. nov. et sp. nov. in the phylogeny of living angiosperms after its addition to the matrices of Kvaček et al.^46^ and Coiro et al.^47^. Color scales indicate all most parsimonious positions in red (MP), and that one step less parsimonious in yellow (MP + 1) and two steps less parsimonious in green (MP + 2).

Our cladistic analysis (Fig. 8) was performed using the matrices by Kvaček et al.^46^ and Coiro et al.^47^ with the D&E backbone from Endress & Doyle^48^. Our results show that the single most parsimonious position of *Notocyamus* is within Proteales, as sister to *Nelumbo* (Nelumbonaceae), and supported by synapomorphies of the two taxa within Proteales, such as the lack of freely ending veinlets (CH14), solitary flowers (CH43), and the presence of pits in receptacle bearing individual carpels (CH52). The herbaceous habit (CH1) and anomocytic stomata (CH37) are also shared between them and could equally be synapomorphies among Proteales since all other taxa (including Sabiaceae) are woody and predominantly possess paracytic and laterocytic stomata (modified to anomocytic in some Platanaceae and Sabiaceae)^49,50^. However, the point on the tree at which these shared character states arose is ambiguous. Chloranthoid teeth (CH36) are a plesiomorphic character widespread in basal eudicots^12^, and their loss is a synapomorphy of Proteales (modified to platanoid teeth in Platanaceae). However, *Notocyamus* and *Nelumbo* still differ in the scoring of cambium (CH6), vessel perforation (CH09), leaf shape (CH30), and base of blade (CH33), and by the presence of paracytic stomata (CH37). In Proteales, loss of cambium (CH6) and evolution of peltate leaves (CH33) are autapomorphies of *Nelumbo*.

The position sister to Magnoliales and Laurales is one step less parsimonious and supported by the fiber pitting (CH10), and the presence of solitary flowers (CH43) (common in Magnoliales). However, they differ markedly from *Notocyamus* and *Nelumbo* in having freely ending veinlets (CH14) (lacking only in Lauraceae), pinnate major venation (CH31) (modified to palmate in a few Laurales), and in lacking pits in the receptacle (CH52).

We also found an alternative two step less parsimonious position in Proteales with *Notocyamus* sister to Platanaceae + Proteaceae. This position is supported by mixed scalariform and simple vessel perforation plates (CH9), but it requires two origins of freely ending veinlets (CH14), solitary flowers (CH43), and receptacle with carpel-bearing pits (CH52), or one origin of these features followed by a reversal in *Platanus* and Proteaceae.

Three additional two step less parsimonious positions were retrieved among magnoliids, with *Notocyamus* sister to either Piperales or *Galbulimima* in Magnoliales, and monocot. The alternate position as sister to Piperales is supported by palmate venation (CH31) but requires one more step in each of these characters (CH14, CH43, CH52) than the position with *Nelumbo*. The position sister to *Galbulimima* is supported by vessel perforation (CH9) and fiber pitting (CH10) but also requires one more step in several characters, in this case due to the woody habit (CH01), vessel grouping (CH11), freely ending veinlets (CH14), pinnate major venation (CH30) and the absence of pits in the receptacle (CH51). The position as sister to monocot is supported by leaf shape (CH30) but requires one more step in each of these characters (CH31, CH43, CH52) than the position with *Nelumbo*.

## Discussion

### Age of the fossil and paleogeographical implications

*Notocyamus* is described from the late Barremian?/Aptian of Brazil and is thus older than the previously oldest representative of its clade, i.e., *Nelumbites extenuinervis* from the middle to upper Albian of USA^18,19^, but also than isolated leaves of *Nelumbites* from Kazakhstan^16^. Its older age is consistent with the fact that several of its morphological characters are more plesiomorphic than those of Albian *Nelumbites* and extant *Nelumbo*. In addition, molecular dating indicates that the divergence time of Nelumbonaceae and Platanaceae is around 83 to 123 Ma^14,19,51–53^. These older age estimates are very close to the first pieces of evidence from the fossil record of this family even though their divergence time could still be earlier^14,54^.

Our findings also raise questions on the paleogeographic origin of the family since until our study, distributions of all previous fossil records suggested an origin of *Nelumbo* in Laurasian mid-latitudes^25^. Also, Ramírez-Barahona et al.^55^ suggest that the ancestral biome of Nelumbonaceae is most likely coupled with more temperate than tropical climates. However, whether their initial evolutionary history is linked to Laurasian mid-latitude areas and ancestral temperate biomes^25,55^, or not, remains unclear. Our report preliminarily suggests that the initial evolution of Nelumbonaceae may have occurred in northern Gondwanan low-latitude areas under a seasonal tropical climate since *Notocyamus* is currently the oldest record of this family and displays traits interpreted as ancestral for the family. This new Brazilian fossil also adds data to postulate that the paleoequatorial tropical areas were an important center of early diversification of basal eudicots in the Early Cretaceous before reaching mid-latitude areas, where they became widespread from the Albian onwards*.* This hypothesis was discussed by Doyle^5^ based on fossil pollen records and is currently being reinforced by an increasing number of macrofossils of basal eudicots described from the Crato Formation^8^, but also by evidence from tricolpate pollen already reported in this same lithostratigraphic unit^32^.

### Morphological adaptation for an aquatic habitat

**Figure 9:**
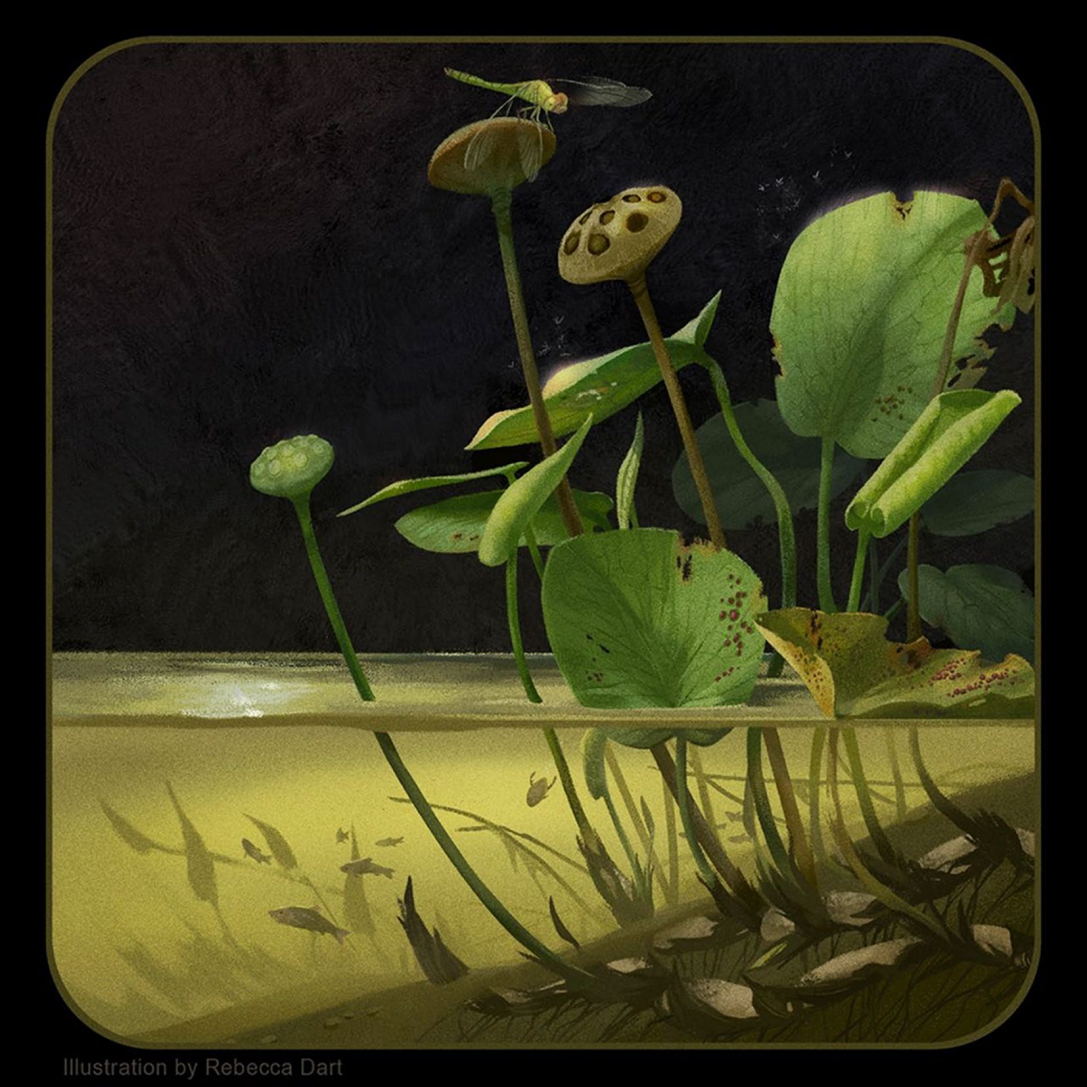
Reconstruction of *Notocyamus hydrophobus* gen. nov. et sp. nov. in its likely environment. Illustration by Rebecca Dart (Vancouver–British Columbia).

In terms of habit and morphology, *Notocyamus* resembles living Nelumbonaceae and implies a paleoecology adapted to similar aquatic conditions (see reconstruction, Fig. 9). In contrast to the woody habit of the other Proteales, fossil and extant members of the family share the same rhizomatous habit^26,56^ (Fig. 1A). In addition, the presence of long petioles in both *Notocyamus* (Figs. 1A and 2A) and living Nelumbonaceae enables the laminae to reach the water surface and beyond. In *Nelumbo*, this elongation is conditioned to water depth and flooding events, and this adaptive response is mediated by ethylene gas flowing through aerenchyma^57,58^. Such a specialized tissue with numerous air chambers is also present in the petiole of *Notocyamus* and living Nelumbonaceae. It also typically occurs in plants growing in aquatic-related environments under anoxic conditions, not only to allow sufficient oxygen levels to maintain respiration through gas canals but also to improve their mechanical support^58–60^.

The leaf epidermises of *Notocyamus* and living *Nelumbo* are also essentially similar by having anomocytic stomata (Fig. 3D,F) with similar density average values on the upper surfaces^15,61^, and dense papillae with superhydrophobic properties (Fig. 3), producing the famous “Lotus effect”^62^. The water-repellent nature of leaves facilitates water and dirt particles removal on the leaf surface and ensures stomata function and gas exchanges^63,64^. Such water-repellent leaf surfaces are especially common in herbaceous plants living in disturbed and wetland environments^63^. The only notable micromorphological difference is that the leaf of *Notocyamus* also bears likely (brachy-) paracytic stomata and distinct epidermal papillae on both sides, whereas those of *Nelumbo* are exclusively anomocytic and almost entirely epistomatic with papillae present only on their adaxial (upper) surfaces^61,65–67^. It suggests the development of emergent leaves in *Notocyamus* and subsequent exaptation of the Lotus effect in peltate leaves of Nelumbonaceae. Such emergent and amphistomatic leaves, despite being rare in *Nelumbo*, are also well-documented in other aquatic plant lineages^59^. In addition, the relatively high leaf vein density (ca. 9 mm^−1^) in *Notocyamus* may support a higher water supply capacity leading to a better leaf hydraulic conductance and consequently higher photosynthetic and transpiration rates^68–70^. As in living *Nelumbo* species, it would probably also allow establishment in sunny habitats under conditions of high temperatures.

Both *Notocyamus* and living Nelumbonaceae also display a typically enlarged floral receptacle that develops into an aggregate fruit with several nutlets embedded in pits^26,71^ (Fig. 1A,D). This kind of fruit is adapted to float in aquatic habitats and disperses its diaspores passively by hydrochory^13,72^. In addition, the reproductive organs associated with though not attached to the leaves of *Nelumbites extenuinervis* Upchurch, Crane et Drinnan, and *Nelumbo puertae* Gandolfo et Cuneo also share an enlarged receptacle with fruitlets^18,23^.

### Comparison with extinct and extant Proteales and evolutionary implications

*Notocyamus* is unambiguously more closely related to Nelumbonaceae than to any other extant family. However, it also displays a unique combination of ancestral and derived characters, some of which are shared with other members of Proteales and may indicate that these structures represent a transitional stage between woody and herbaceous protealeans.

In terms of leaf architecture, the lobed and/or toothed margins common in almost all leaves of *Platanus* L. (Platanaceae) are unknown in *Notocyamus*, but both have similar palinactinodromous venation and marginal lamina attachment (Figs. 1A,C and 2A,C), as does *Bellendena* R.Br. (Proteaceae)^73,74^, suggesting that these traits were homologous in all these groups. This similarity is also seen in some Cretaceous platanoid leaves assigned to *Credneria* Zenker and *Platanus*, which also display palinactinodromous venation^75^. *Notocyamus* often shares the obovate to elliptical leaf shapes (Figs. 1A and 2A) with these fossils, and related lineages such as Proteaceae and Sabiaceae, which contrasts with the leaves of *Nelumbo* and its younger fossil relatives, indicating that Nelumbonaceae passed through this morphological stage before having orbicular leaves^20^.

The presence of paracytic and anomocytic stomata in *Notocyamus* may strengthen the concept of its mosaic of ancestral and derived characters. Since almost all woody protealeans vary between paracytic and laterocytic stomata, and *Nelumbo* has exclusively anomocytic stomata, this may suggest that *Notocyamus* is in a transitional form between ancestral (paracytic stomata) and derived (anomocytic stomata) anatomical traits.

If the leaf architecture of *Notocyamus* is considered to be plesiomorphic for the family, *Notocyamus* can be interpreted as a first evolutionary step towards *Nelumbo*. *Nelumbites*^18^, from the Albian, can be interpreted as a second evolutionary step. Its leaf blade is already peltate, but eccentric, its primary venation is actinomorphic but comprises only a few primaries (< 10), and its lamina is orbicular, displaying a basal indentation reminiscent of the sometimes-cordate leaf bases of *Notocyamus*. *Exnelumbites*^20^, from the Campanian–Maastrichtian, which can be interpreted as a third evolutionary step, is already centrally peltate but displays 12–13 primaries and surprisingly bears chloranthoid teeth. *Paleonelumbo*, from the Paleocene, is another genus with distinct leaf architecture: although its leaves are centrally peltate, they are quite distinct from those of *Nelumbo* in being lobed and having 10–15 primary veins^15,20^. *Nelumbago*, also from the Paleocene, is, however, very similar to extant and extinct species of *Nelumbo* in terms of foliar architecture and can therefore be interpreted as the next to last evolutionary step in having centrally peltate and entire-margined leaves with > 20 bifurcating primary veins but still no central disk^15,20^. All extinct and extant Nelumbonaceae cited above suggest that peltation of their leaves did not evolve until a certain time after the divergence of Nelumbonaceae.

Despite differing significantly in its leaf architecture by having a distinct primary venation type, *Notocyamus* exhibits a special similarity to *Nelumbo* in lacking the freely ending veinlets. This leaf character is rare in basal eudicots and occurs only in Nelumbonaceae in Proteales. In addition, *Notocyamus* and *Nelumbo* share the same entire-margined leaf blades, bifurcating primary venation, festooned brochidodromous secondary venation, tertiary and quaternary veins with mixed course, and six-sided areolae^15,20^.

In terms of reproductive structures, *Notocyamus* and other Nelumbonaceae both produce single flowers sharing an apomorphic feature: carpel-bearing pits in the receptacle. The shape of the floral receptacle appears to have evolved from ellipsoidal in *Notocyamus*, to slightly conical in *Nelumbites extenuinervis* and *Nelumbo puertae*^18,23^, culminating in the well-known obconical shape in *Nelumbo*. Although the reproductive organs of both *Notocyamus* and *Nelumbo* are borne on long peduncles (Fig. 1A), their peduncles are quite distinct anatomically^76^, because only *Notocyamus* has secondary xylem produced by a vascular cambium, as in other members of Proteales, which are woody plants. Interestingly, the secondary growth did not disappear in the nelumbonaceous line until some time after its divergence from other Proteales. In its peduncle, *Notocyamus* bears several water-conducing cells in each primary xylem bundle, and in the secondary xylem (wood) nonconductive cells (fibers with lignified walls) occur (Figs. 5 and 6). Most woody tissues of *Notocyamus* consist of fibers with simple and slit-like pits (Figs. 5 and 6D). These structures also occur in the most closely related lineages: Platanaceae usually have fiber tracheids with bordered and simple pits^77^, most genera of the Proteaceae have libriform fibers with simple and bordered pits^78^, and the majority of Sabiaceae taxa have libriform fibers with vestigial bordered pits^79^. Concerning the type of vessel perforation in the primary xylem, *Notocyamus* displays the same combination of simple and scalariform vessel perforation plates as Platanaceae, Proteaceae, and Sabiaceae, suggesting that this state could be a synapomorphy of the Proteales, but *Nelumbo* may represent a reversal of this trend. The specialization of vessel perforation plates from scalariform to simple has long been interpreted as derived and occurred several times in different lineages^80,81^.

### Overall importance for evolution of Proteales and conclusions

Fossils of Proteales show that members of this group already inhabited contrasting environments during the Early Cretaceous, with terrestrial woody Platanaceae and aquatic herbaceous Nelumbonaceae. Unfortunately, macrofossils of Proteaceae and Sabiaceae are sporadic in the Cretaceous and better recognized and described in Cenozoic floras^9^. However, fossil representatives of Nelumbonaceae and Platanaceae confirm that huge differences in gross morphology already existed between Cretaceous members of these families (e.g., *Nelumbites*, and *Sapindopsis*, respectively). In addition, the fossils show that the living *Platanus* and *Nelumbo* are similar to some of their Early Cretaceous members, and that these clades thus likely experienced early rapid morphological evolution followed by a long period of relative stasis.

Besides our results from cladistics supporting the position of *Notocyamus* as a stem-relative of Nelumbonaceae, the fossils not only push back the minimum age of the appearance of the lotus lineage to at least the early Aptian but also shed light on the morphological evolution of its vegetative and reproductive traits. Despite the putative stasis previously reported in this family, their stem lineages may still have undergone considerable morphological changes throughout geological time. In addition, our report surprisingly provides new lines of evidence that the divergence between Nelumbonaceae and other members of Proteales may have occurred in a stepwise fashion. Compared to other extinct nelumbonaceous fossils, *Notocyamus* displays a new and unique mosaic of ancestral states shared with Platanaceae and Proteaceae, and derived states shared with *Nelumbo*, and clarifies important evolutionary steps of Nelumbonaceae and Proteales as a whole. Thus, *Notocyamus* ideally fills the morphological gap between aquatic Nelumbonaceae and their most recent common ancestor with woody Proteales, and can therefore be seen as one of those “missing links” that are rarely preserved in the fossil record.

## Methods

### Fossil analysis

The fossils were studied using a Leica Wild M10 microscope equipped with a Leica DFC 425 camera. Overview images were taken with a Canon 250D camera. Vegetative structures (leaves, petiole, and peduncle) of *Notocyamus* exhibit anatomical details preserved by iron oxide replacement of tissues. These details were observed in a high vacuum with a Scanning Electron Microscope (SEM) (Carl Zeiss EVO 50 and EVO LS 10, Germany). For SEM analyses, pieces of leaves, petiole and peduncle were removed from the specimen, mounted on stubs, and coated with gold for four minutes with a Polaron SC7640 sputter coater. Photographs of fossils were edited with Adobe Photoshop software.

### Leaf architecture

The description of leaf architecture follows Ellis et al.^45^.

### Leaf width inferences

The leaf width measurements of *Notocyamus* (holotype) were inferred from leaves with at least half the lamina preserved (including folded leaves), multiplying the distance from the midvein to the lamina margin by two supposing a symmetrical lamina shape. They were used to estimate the laminar size, L:W ratio, and leaf shape.

### Vein and stomata density measurements

The vein density (mm of vein per mm^2^) of *Notocyamus* was calculated based on an SEM image (Fig. 3B) that best shows the length of quinternary veins. The measurements were made using the program ImageJ (https://imagej.nih.gov/).

The stomatal density (SD) on leaves of *Notocyamus* was calculated using seven SEM images from different leaf pieces on both adaxial and abaxial sides. The presence of stomata was counted on squares of 0.12 to 0.3 mm^2^ to calculate the stomata density in mm^−2^.

### Fruitlet volume measurements

The volume of fruitlets was estimated based on the formula V = 4/3πr^3^, following Tiffney^82^.

### Thin sections

Petrographic thin sections of the peduncle were made in the three standard sections used for studying wood anatomy: transversal, radial, and tangential (sensu Jones and Rowe^83^). Slides were analyzed and photographed using a Zeiss light microscope (Axioscope 5) coupled to a Canon 60D camera.

### Cladistic analysis

The phylogenetic analysis was performed by adding the fossil as a new taxon to the morphological data set from Kvaček et al.^46^ and Coiro et al.^47^ with character states presented in Supplementary Information, using the D&E backbone constraint tree^48^. The relative parsimony of all positions of the fossil was evaluated using Mesquite 3.61^84^, and the number of required character state changes (steps) in each alternative tree was recalculated. All characters were unordered and equally weighted.

### Updated age estimation for the Crato Formation

The Crato Formation, the second lithostratigraphic unit from the base of the Santana Group, has been repeatedly dated as upper Aptian age based on palynological evidence^32^. By contrast, new results of Melo et al.^34^, Barreto et al.^36^ and Arai & Assine^85^ consistently revealed an upper Aptian age for the Romualdo Formation, the uppermost unit of the Santana Group, based on relative and absolute dating methods. In addition, the Ipubi Formation, which underlies the Romualdo Formation, contains two important layers for age estimation, the black shale at its base and the evaporite layer soon above. Lúcio et al.^33^ dated this evaporite layer by Re–Os isotope studies as 123 ± 3.5 Ma (126.5 to 119.5 Ma). Besides, isotope and organic geochemistry of this black shale provides strong evidence that it was deposited during the global Ocean Anoxic Event OAE-1a^35^. The numerical age of the base of OAE-1a, called the Selli event, is ca. 119–120 Ma^86^. Thus, the Crato Formation, underlying the Ipubi Formation, is at least older than 120 Ma, which is not far above the base of the Aptian, or even older if there was a hiatus between the formations^27^.

Since 2022, the updated international chronostratigraphic chart shows a new age for the base of the Aptian (121.4 Ma instead of 125 Ma), which is correlated with a short-lasting magnetostratigraphic zone^87^.

According to Neumann et al.^29^, the C6 carbonate unit of the Crato Formation could represent a period of up to 0.5 Ma. Altogether, the Crato limestones C1–C6 could likely represent several million years, which would extend its absolute age range towards the upper Barremian. However, such a fundamental change in the age of the Crato Formation needs to be supported by additional unambiguous evidence in future publications.

## Supplementary Information

Supplementary Information.

## Data Availability

All data analyzed during the current study are included in this published article and its supplementary information file.
