# Supplementary material for: A new remarkable Early Cretaceous nelumbonaceous fossil bridges the gap between herbaceous aquatic and woody protealeans
Source: Sci Rep. 2023 Jun 2;13:8978. doi: 10.1038/s41598-023-33356-z (PMC10238487; doi:10.1038/s41598-023-33356-z)
Supplement: Supplementary file 1 — Supplementary Information. [file 41598_2023_33356_MOESM1_ESM.docx]

**Supplementary information**

**Character coding (following Kvaček *et al*., 2016 and Coiro *et al*., 2020).** Character states coded for the fossil are in bold.

**Characters**

1 Habit (0) tree or shrub, **(1) rhizomatous, scandent, or acaulescent.**

2 Stele **(0) eustele**, (1) (pseudo)siphonostele, (2) monocot-type

(atactostele).

3 Inverted cortical bundles **(0) absent**, (1) present.

6 Cambium **(0) present**, (1) absent.

7 Storied structure (in tracheids and axial parenchyma, phloem) **(0)**

**absent**, (1) present. Scored as unknown (?) when secondary growth is

nearly or entirely lacking.

8 Tracheary elements (0) tracheids and elements with porose pit membranes,

**(1) vessel members with typical perforations.**

9 Vessel perforations (end-wall pits in vesselless taxa) (0) scalariform,

**(1) scalariform and simple in the same wood,** (2) simple.

10 Fiber pitting (lateral pitting of tracheids in vesselless taxa) (0) distinctly

bordered, **(1) minutely bordered or simple**. Scored as unknown

when secondary xylem is absent or fibers are replaced by pervasive

parenchyma.

11 Vessel grouping **(0) predominantly solitary**, (1) mostly pairs or

multiples.

12 Rays **(**0) narrow (generally not more than four cells wide), **(1) wide.**

14 Freely ending veinlets **(0) absent**, (1) present.

22 Phyllotaxis **(0) alternate (spiral or distichous),** (1) opposite or

whorled.

29 Leaf blade **(0) bifacial**, (1) unifacial.

30 Leaf shape **(0) obovate to elliptical to oblong**, (1) ovate, (2) linear.

31 Major venation (0) pinnate with secondaries at more or less constant

angle, (**1) palmate (actinodromous or acrodromous**) or crowded (pinnate

with crowded basal secondaries, upward decreasing angle), (2)

parallel (lateral veins departing at low angles from the midrib and

converging and fusing apically).

32 Fine venation **(0) reticulate**, (1) open dichotomous in some or all

leaves.

33 Base of blade **(0) not peltate**, (1) peltate in some or all leaves.

34 Apex of blade **(0) simple**, (1) bilobed.

35 Leaf dissection **(0) simple**, (1) some or all leaves lobed or compound.

36 Marginal teeth **(0) absent,** (1) chloranthoid, (2) monimioid, (3)

platanoid.

37 Stomata (predominant type on leaf) **(0) paracytic**, (1) laterocytic, **(2)**

**anomocytic (including actinocytic),** (3) stephanocytic (including

cyclocytic and tetracytic).

39 Palisade parenchyma (0) absent (mesophyll homogeneous), **(1) present**

**(mesophyll dorsiventral).**

43 Inflorescence **(0) solitary flower (or occasionally with 1–2 lateral**

**flowers**), (1) botryoid, panicle, or thyrsoid (monotelic), (2) raceme,

spike, or thyrse (polytelic). In taxa with unisexual flowers, scoring is

based on the sex with the more complex inflorescences.

44 Inflorescence partial units **(0) single flowers**, (1) cymes.

45 Inflorescence (or partial inflorescence) **(0) not modified**, (1) modified

into globular head.

46 Pedicel **(0) present in some or all flowers**, (1) absent or highly reduced

(flower sessile or subsessile).

50 Floral base **(0) hypanthium absent, superior ovary,** (1) hypanthium

present, superior ovary, (2) partially or completely inferior ovary.

51 Floral receptacle (female portion) **(0) short**, (1) elongate.

52 Pits in receptacle bearing individual carpels (0) absent, **(1) present.**

54 Floral **apex (0) used up after production of carpels**, (1) protruding in

mature flower. Unicarpellate taxa scored as unknown.

108 Carpel fusion **(0) apocarpous**, (1) parasyncarpous, (2) eusyncarpous

(at least basally). Taxa with one carpel scored as unknown

(inapplicable).
